# Appetite for Destruction: A Psychometric Examination and Prevalence Estimation of Destructive Leadership in Sweden

**DOI:** 10.3389/fpsyg.2021.668838

**Published:** 2021-08-06

**Authors:** Robert Lundmark, Andreas Stenling, Ulrica von Thiele Schwarz, Susanne Tafvelin

**Affiliations:** ^1^Department of Psychology, Umeå University, Umeå, Sweden; ^2^Department of Sport Science and Physical Education, University of Agder, Kristiansand, Norway; ^3^School of Health, Care and Social Welfare, Mälardalen University, Västerås, Sweden

**Keywords:** destructive leadership, abusive supervision, leadership profiles, leadership measure, prevalence

## Abstract

There is a growing awareness that destructive leadership has a significant negative impact on employe outcomes. However, little is known about the content and dimensionality of this multidimensional concept, and there are few reliable measures available for organizations and researchers to evaluate these behaviors. Based on a representative sample (*N* = 1132) of the Swedish workforce, the aim of this study is threefold: first, to examine the factor structure and validity of an easy-to-use multidimensional destructive leadership measure (Destrudo-L)in the general Swedish work context; second, to identify destructive leadership profiles using latent profile analysis (LPA), and determine in what way they are related to employe outcomes; third, to examine the prevalence of destructive leadership using population weights to estimate responses of a population total in the Swedish workforce (*N* = 3100282). Our analysis supported the structural validity of Destrudo-L, reflecting both a global factor and specific subdimensions. We identified seven unique destructive leadership profiles along a passive and active continuum of destructive leadership behaviors, with the active showing a less favorable relation to employe outcomes. Finally, we found that a substantial proportion of the Swedish workforce report being exposed to destructive leadership (36.4–43.5%, depending on method used). Active destructive leadership was more common in the public sector and passive destructive leadership in the private. Given the potentially severe effects and the commonness of these behaviors, we argue that organizations should work actively with strategies to identify and intervene, to prevent and to handle the manifestation of these harmful behaviors.

## Introduction

Leadership research has, over the two last decades, increasingly paid attention to the “dark” side of leadership (Mackey et al., 2019). To capture the range of these adverse behaviors, destructive leadership is commonly used as an overarching expression (Krasikova et al., 2013). The main reason for the growing interest in studying destructive leadership is the potential negative effects of such behaviors for employes and organizations (Schyns and Schilling, 2013; Zhang and Liao, 2015; Fors Brandebo et al., 2016). The severity of the problem has been highlighted by indications from a prevalence study, which showed that destructive leadership behaviors are quite common (Aasland et al., 2010).

Most researchers agree that destructive leadership includes several dimensions (Skogstad et al., 2017; Schmid et al., 2018). However, little is still known about whether these dimensions also form, and can be understood, in terms of a global destructive leadership factor with subdimensions, or if they are, in fact, distinct behavioral clusters (Tepper, 2007; Larsson et al., 2012). Likewise, there is a lack of knowledge concerning differences and similarities in the associations among the dimensions of destructive leadership, employe, and organizational outcomes (Mackey et al., 2019; Trépanier et al., 2019). Due to different views on how to conceptualize and define destructive leadership, prevalence of destructive leadership behaviors at work remains uncertain (Schyns and Schilling, 2013; Schmid et al., 2018; Thoroughgood et al., 2018).

Successfully resolving these gaps in the literature demands the development of well-validated and reliable measures that can capture the essence of the multidimensionality of destructive leadership (Shaw et al., 2011; Thoroughgood et al., 2012; Krasikova et al., 2013). The most commonly used measure to date, Tepper (2000) 15-item abusive supervision scale, does not discriminate between different types of behaviors, and has shown varied psychometric qualities (Tepper, 2007; Mackey et al., 2019). Other measures focus on one or several specific aspects of destructive leadership, but not as subdimensions of a global destructive leadership factor (Aasland et al., 2010), and some are very extensive in terms of the number of questions (Shaw et al., 2011). Additionally, some measures have examined a global destructive leader factor (Thoroughgood et al., 2012), but include dimensions beyond what are commonly considered destructive leadership behaviors (Schyns and Schilling, 2013). Thus, with a well-validated and reliable measure that captures the multidimensionality of destructive leadership, we can advance our understanding of destructive leadership as a concept. This, in turn, allows us to examine how these dimensions are associated with different outcomes, and how common destructive leadership is overall, and in terms of different dimensions.

Based on the above, the aim of the present paper is threefold: first, to examine the structural validity of a multidimensional destructive leadership measure, Destrudo-L (Larsson et al., 2012). Destrudo-L is a measure that incorporates several dimensions of destructive leadership and was initially developed for use in a Scandinavian military setting (Larsson et al., 2012; Fors Brandebo et al., 2016). We explore whether it is also applicable as a measure to evaluate destructive leadership behaviors in a general work context. Additionally, we test the convergent and discriminant validity of Destrudo-L in relation to a variety of outcome measures and other leadership scales, moving beyond previous psychometric evaluations of the measure (Larsson et al., 2012).

Second, we aim to examine whether it is possible to identify different destructive leadership profiles based on the subdimensions of the measure, and whether they are related to outcomes in the same or in different ways. We thus explore which kinds of destructive leadership profiles are identifiable and how these profiles relate to different employe well-being and performance outcomes. In so doing, we add to previous studies that have investigated behavioral clusters of destructive leadership behaviors, but without relating such clusters to outcomes (Aasland et al., 2010; Shaw et al., 2011). Thereby, the present study is the first to relate latent profiles of destructive leadership to employe outcomes.

Third, we aim to examine the prevalence of destructive leadership behaviors using a representative sample of the Swedish workforce. A few prevalence studies on destructive leadership behaviors have been performed in other countries (i.e., Norway: Aasland et al., 2010; United States: Schat et al., 2006; Netherlands: Hubert and van Veldhoven, 2001). However, due to variation in operationalization and evaluation methods used, prevalence numbers remain uncertain (Schyns and Schilling, 2013). Using several methods and reporting on different dimensions of destructive leadership, we make comparisons to previous prevalence examinations. We also examine differences between the public and private sector, going beyond previous studies. Moreover, the current study is the first to examine prevalence of destructive leadership using latent profiles of a multi-dimensional measure with a global destructive leadership factor. Further, by studying the prevalence of various destructive leadership behaviors included in Destrudo-L, our study contributes with an in-depth examination of behaviors that occur more or less frequently. The profile analysis also helps determine the commonness of disparate destructive leadership behaviors. Thereby, in combination with studying the various dimensions and profiles associated with outcomes, this can help guide future intervention efforts.

### The Concept of Destructive Leadership

Leadership is a multifaceted and continuously evolving research field (Clark and Harrison, 2018), and the sub-field of destructive leadership, which has evolved in parallel to post-heroic leadership research, is no exception (Mackey et al., 2019). The interest in destructive leadership is mainly based on the extent and severity to which these behaviors influence employe outcomes negatively (Zhang and Liao, 2015). The emphasis on destructive leadership as consequential for employe outcomes is also reflected in the commonly used definition of destructive leadership, which refers to behaviors that undermine employe motivation, well-being, or job satisfaction (Einarsen et al., 2007). Over the last two decades, discussions on what constitutes destructive leadership has developed from merely including leaders’ aggressive behaviors toward employes to taking a wider perspective (Skogstad et al., 2017). A wider perspective involves a more holistic approach in which leaders’ passive behaviors, susceptible followers, and the environment, also contribute to the understanding of the destructive leadership process (Thoroughgood et al., 2018).

Currently, researchers seem to agree that destructive leadership is a multidimensional phenomenon (Skogstad et al., 2017). However, there is still some debate on the dimensions encompassed by the concept (Thoroughgood et al., 2012). For example, the commonly used definition developed by Einarsen et al. (2007) has been repeatedly criticized for its inclusion of the supportive-disloyal dimension (i.e., organizational directed leadership behaviors) of destructive leadership (Krasikova et al., 2013; Schyns and Schilling, 2013). Leadership literature generally suggests that as leadership in the workplace involves employe-targeted influence, it should not include non-employe targeting behaviors (e.g., counter-productive work behaviors such as supportive-disloyal behaviors; Schyns and Schilling, 2013). Consequently, a recent study on the differential effects of both employe- and organizational-directed destructive leadership shows that negative employe effects are produced primarily by the former (Schmid et al., 2018).

An employe-oriented perspective on the definition of destructive leadership is also valid from a theoretical perspective. For example, Conservation of Resources (COR; Hobfoll, 1989) has been suggested as a useful framework to understand how destructive leadership may influence employe outcomes (e.g., Otto et al., 2018). COR depicts that objects (e.g., tools), personal characteristics (e.g., emotional stability), conditions (e.g., social support), or energies (e.g., money) are valued resources that employes’ strive to obtain, retain, foster and protect (Hobfoll and Shirom, 2001). The theory further proposes loss of resources as the defining principle for stress. Hence, stress ensues either if individuals perceive a threat to resources, actual loss of resources, or if they are unable to regain a resource loss (Hobfoll, 1989). As the quality of the social (i.e., leader-employe)relationship is understood as a major resource, destructive leadership behaviors places employes at risk for high levels of stress. In turn, this may negatively influence a range of employe well-being (e.g., in terms of job satisfaction) and performance (e.g., in terms of work-role performance) outcomes (Inceoglu et al., 2018).

We agree with the view of leadership at work as a social process, and that therefore non-employe targeted behaviors should not be included in the definition of destructive leadership. However, whereas some exclude passive/indirect leadership behaviors from their definition of destructive leadership (Schyns and Schilling, 2013), we share the view that the destructiveness of a leadership process should be determined by the actual harm it causes employes (Skogstad et al., 2007; Thoroughgood et al., 2018). That is, not by the extent to which a leader’s destructive behaviors are intentional or perceived as expressions of aggressiveness by employes. Such a view is in line with empirical findings of the kinds of behaviors employes describe as destructive leadership behaviors when interviewed (Schilling, 2009; Shaw et al., 2011; Larsson et al., 2012). In other words, forms of more or less active/direct, as well as passive/indirect employe-directed leadership behaviors are included in employes’ appraisals. It is also in line with theoretical suggestions on what kind of leadership behaviors that may cause negative employe outcomes. In addition to explain negative employe outcomes of active destructive leadership, COR theory has also been used to explain the relation between passive destructive leadership and negative employe outcomes. From an employe perspective, passive destructive leadership stands in the way of guidance and social support needed to perform job tasks satisfactory, which may cause resource depletion and thereby be a cause of workplace stress (Einarsen et al., 2007).

Hence, with few exceptions (e.g., Thoroughgood et al., 2012), most recent studies also exclude non-social behaviors, and organize destructive leadership behaviors into two main categories: active/direct and passive/indirect (e.g., Fosse et al., 2019; Kaluza et al., 2019; Trépanier et al., 2019; Lundmark et al., 2020).

### Multidimensional Measurement of Destructive Leadership

There are few easy-to-use measures to evaluate the multidimensional nature of destructive leadership (Krasikova et al., 2013). Destructive leadership measures are either very extensive (Shaw et al., 2011), or combine a multitude of separate scales to capture different dimensions (Aasland et al., 2010; Lundmark et al., 2020), but without exploring the potential existence of a global factor (Larsson et al., 2012). An exception is the measure developed by Thoroughgood et al. (2012). However, they include organizationally directed deviant behaviors (e.g., steeling), as well as sexual harassment behaviors (e.g., “brings inappropriate material to work”). Thus, although including a global factor with distinct subdimensions, it covers a wider range of leaders’ workplace deviance, moving beyond the core of the destructive leadership definition as employe-directed behaviors. The only measure we have found that aligns with an employe-directed approach to destructive leadership, includes both passive and active destructive leadership aspects, is easy to use (i.e., not too long), and includes a global factor with subdimensions is Destrudo-L (Larsson et al., 2012).

Destrudo-L consists of five subdimensions of destructive leadership: *arrogant/unfair, threats/punishments/overdemands, ego-oriented/false, passive/cowardly*, and *uncertain/unclear/messy* (Larsson et al., 2012). The five factors are translatable into the labels or dimensions of destructive leadership that other researchers have used in their conceptual work (e.g., Tepper, 2000; Einarsen et al., 2007; Skogstad et al., 2007; Schilling, 2009). The subdimensions arrogant/unfair and threats/punishments/overdemands in Destrudo-L describe behaviors similar to those described by a tyrannical leadership, as suggested by Einarsen et al. (2007). The ego-oriented/false subdimension, on the other hand, resembles what Einarsen et al. (2007) call a derailed leadership. Finally, the passive/cowardly and uncertain/unclear/messy subdimensions consider more passive/indirect leadership behaviors, comparable to the laissez-faire, avoidant, or failed subdimensions of destructive leadership suggested by Skogstad et al. (2007). Thus, this indicates that on a conceptual level, there is substantial overlap between the subdimensions of Destrudo-L and subdimensions identified in literature on destructive leadership (for a full compilation, see Larsson et al., 2012).

There are two important sources of construct-relevant multidimensionality to consider in relation to constructs that are hierarchical, with subdimensions conceptually related to each other (such as the subdimensions of Destrudo-L). First, it is necessary to consider the hierarchical nature, by acknowledging the presence of an overarching global factor, which can be identified using bifactor models (Morin et al., 2016). Second, most items are, to some degree, imperfect and have some systematic association with other constructs, a phenomenon referred to as the fallible nature of indicators (Morin et al., 2016). Therefore, it is typically possible to justify cross-loadings based on substantive theory or item content in multidimensional measures (Asparouhov and Muthén, 2009). In the independent clusters model confirmatory factor analysis (ICM-CFA), cross-loadings are constrained to zero, which often inflates and biases factor correlations and worsens the construct validity of the factor structure (Marsh et al., 2014). In exploratory structural equation modeling (ESEM), all cross-loadings are estimated and assumed to be close to zero but not exactly zero, which takes into account the fallible nature of indicators, reduces factor correlations, and improves discriminant validity (Marsh et al., 2014; Morin et al., 2016). In line with previous recommendations (e.g., Marsh et al., 2014), we compare ICM-CFA models with ESEM models to identify the model that provides the best fit to the data.

As Destrudo-L was developed primarily for use in military settings, neither its psychometrical properties nor its relations with outcomes has been tested in a general work context. However, there have been calls to examine its reliability and validity in a general work context (Larsson et al., 2012). Additionally, there have been no examinations of the convergent and discriminant validity of the Destrudo-L, nor has its suggested factor structure, with one global factor and five subdimensions, ever been replicated.

Research question 1: Can the factor structure of Destrudo-L, with a global destructive leadership factor and specific subdimensions, be replicated using a representative work sample?

Research question 2: Does Destrudo-L show convergent and discriminant validity, in relation to other measures of adverse leadership (i.e., abusive supervision), constructive leadership (i.e., transformational leadership), and to employe outcomes?

### Destructive Leadership Profiles and Employe Outcomes

Beyond finding effective ways to evaluate destructive leadership, there have been calls to find out more about the unique and relative association between different forms of destructive leadership behaviors and employe outcomes (Schmid et al., 2018). To articulate a meaningful multidimensional understanding of destructive leadership, it is important to know how destructive leadership behaviors are expressed in practice and how these expressions relate to outcomes. Such knowledge can help detect and guide interventions on these behaviors in organizations (Schmid et al., 2018). One way to further explore the dimensionality, the co-occurrence of different subdimensions, and the ways that different dimensions of a destructive leadership relate to outcomes is to use person-oriented approaches, such as cluster analysis or latent class/profile analysis (Aasland et al., 2010; Shaw et al., 2011).

Profile analysis is a relatively new concept within destructive leadership research; the studies by Aasland et al. (2010) and Shaw et al. (2011) have led the way. In their study, Aasland et al. (2010) based the latent classes/profiles on their four suggested dimensions of destructive leadership: Tyrannical, Derailed, Supportive-disloyal, and Laissez-faire. They identified six meaningful latent classes, and determined that (except for a non-destructive cluster) classes dominated by laissez-faire were by far most common. In their cluster analysis, Shaw et al. (2011) combined their overall categorization of leaders as bad or not bad leaders with 22 destructive leadership factors. Shaw et al. (2011) found seven meaningful clusters. They found six meaningful clusters using the same 22 factors in a second study with another sample (Shaw et al., 2014). Based on the results of these studies, Shaw et al. (2011, 2014) concluded that most of the leaders could not be described as destructive in an overall sense, but rather in some part (using a few destructive behavioral categories).

In Destrudo-L, it is possible to understand the global factor of destructive leadership as respondents’ generalized “the leader is, or is not, bad” evaluations (Larsson et al., 2012). Therefore, using an exploratory approach similar to that of Shaw et al. (2011), but combining the global factor and the result of the different subscales, can help reveal how destructive leadership is perceived from the employe perspective.

Previous studies using clustering or latent class/profile approaches did not relate the clusters to outcomes (Aasland et al., 2010; Shaw et al., 2011, 2014). Thus, whether and how clustering contributes to our understanding of the relations between destructive leadership and outcomes is still unknown. Shaw et al. (2011) suggested that only in some clusters could the leader been seen as truly destructive, based on the overall judgment of employes. In evaluating the profiles, we therefore suggest that one way to examine the importance of the overall judgment is to determine whether the higher-order factor is decisive for the profiles’ relation with outcomes.

Research question 3: What meaningful profiles of destructive leadership can be identified in our sample using Destrudo-L?

Research question 4: How are the identified latent profiles related to employe outcomes?

### Prevalence of Destructive Leadership Behaviors

The prevalence of destructive leadership is often used as an argument favoring the study of the phenomenon (e.g., Schyns and Schilling, 2013). However, only one study (Aasland et al., 2010) has examined its prevalence as a multidimensional phenomenon. A few other studies have conducted prevalence research on manager aggression (Hubert and van Veldhoven, 2001; Schat et al., 2006), which, from a multidimensional perspective, can be considered as one aspect of destructive leadership.

Aasland et al. (2010) study established the prevalence of destructive leadership as 83.7% (overall prevalence – exposure of any kind), 33.5% (using operational class method; OCM – cut-off criteria were “often”), and 61% (using latent class cluster; LCC – sum of respondents in destructive clusters) in a Norwegian sample. Schat et al. (2006) studied the overall prevalence of workplace aggression among mangers using six questions in a weighted representative U.S. sample. They found an overall prevalence of 13.5%. Hubert and van Veldhoven (2001) used only one question to evaluate exposure to managers’ aggression, and with an OCM criterion of “sometimes,” rendering a prevalence of 9% in a large Dutch sample. Thus, the subject of study (i.e., what kind of leadership or managerial behaviors), and the methods used to conduct the study, seem to influence the results quite substantially.

Results from a meta-analysis show that constructive (i.e., transformational) leadership is more common in the public sector than in the private sector (Lowe et al., 1996). These results have been explained mainly by the fact that leaders in the public sector are more limited in terms of motivational tools (e.g., rewards), and instead, must rely more on normative appeals and creating an attractive vision (An et al., 2019). However, there has been no such comparison of prevalence rates between the public and private sectors in terms of destructive leadership.

Research question 5: What is the prevalence of destructive leadership behaviors in a Swedish work context?

Research question 6: Are there any differences in the prevalence of destructive leadership behaviors between the public and private sector?

## Materials and Methods

### Participants and Procedure

We used Statistics Sweden, a government agency that produces official statistics, to draw a stratified random sample, representative of the Swedish workforce, based on the Swedish Occupational Register in Sweden. We invited 3000 employes to participate in our study, 750 from each of the four sectors in Swedish working life: private, municipal, state, and government. A paper survey, which took around 20 min to complete, was distributed by mail to the respondents’ home addresses. After three reminders, we received 1132 responses, yielding an overall response rate of 39%. Of the participants, 218 were employed in the private sector, 329 by a municipality, 273 by the state, and 312 by the region. The sample had an average age of 48 years, 66% of which were female, and the respondents had an average tenure in their organization of 13 years.

For the prevalence analysis, we used population weights, provided by Statistics Sweden, to estimate responses of a population total (*N* = 3100282). The population weights compensate for sampling error, non-response error, and coverage error (Lundström and Särndal, 2001). The weights were constructed based on the total number of respondents for the entire survey (*N* = 1132), however the response rate for the Destrudo-L scale was somewhat lower (*n* = 1121). Consulting Statistics Sweden, we therefore estimated these missing responses (*n* = 11 in actual sample, *n* = 23521 in weighted sample) based on average scores on each item of the total sample. To control for potential differences caused by the method used for estimation, we compared results based on the total weighted sample with results based on the weighted sample without those (*n* = 11; *n* = 23521) that were missing. No differences were found on a 1.0% level, and we therefore based all prevalence analysis on the total weighted sample.

### Measures

#### Destructive Leadership

Destructive leadership was measured with the 20-item Destrudo questionnaire (Larsson et al., 2012). The measure includes five subscales with 4 items each: arrogant/unfair (e.g., “is unpleasant”), threats/punishments/overdemands (e.g., “shows violent tendencies”), ego-oriented/false (e.g., “takes the honor of subordinates’ work”), passive/cowardly (e.g., “do not dare to confront others”), uncertain/unclear/messy (e.g., “shows insecurity in his/her role”). The items were rated on a 6-point response scale ranging from 1 (*never/almost never*) to 6 (*always*). Omega (ω) reliability of the scale was 0.958, 95% bootstrap CI [0.953, 962].

We validated the destructive leadership measure against the following leadership measures:

#### Abusive Supervision

As this is the most commonly used measure of adverse leadership behaviors (Mackey et al., 2019). Abusive supervision was assessed with a 15-item scale aimed to capture both passive and active non-physical abuse (Tepper, 2000; Mitchell and Ambrose, 2007). Sample items are “Ridicules me” and “gives me the silent treatment.” Ratings were made on a 6-point scale from 1 (*never/almost never*) to 6 (*always*). Omega (ω) reliability of the scale was 0.940, 95% bootstrap CI [0.928,0.950].

#### Transformational Leadership

As this is the most widely used measure of a constructive leadership (Gardner et al., 2010). Transformational leadership was assessed with a 7-item measure (Carless et al., 2000), An example item is “Communicates a clear and positive vision of the future.” Ratings were made on a five-point response scale ranging from 1 (*never/almost never*) to 5 (*always*). Omega (ω) reliability of the scale was 0.941, 95% bootstrap CI [0.935,0.946].

We validated the destructive leadership measure against the following outcome measures, which have all been concluded to longitudinally relate to destructive leadership (for compilations of previous research see for example: Schyns and Schilling, 2013; Inceoglu et al., 2018):

#### Organizational Citizenship Behaviors

Organizational citizenship behaviors was assessed with the 5-item helping subscale (Ehrhart and Naumann, 2004). An example item is “Always ready to lend a helping hand to other employes around me.” Ratings were made on a 7-point response scale ranging from 1 (*do not agree*) to 7 (*agrees completely*). Omega (ω) reliability of the scale was 0.868, 95% bootstrap CI [0.852,0.882].

#### Turnover Intention

Turnover intention was measured with a 4-item scale (Rudman et al., 2014). An example item is “I consider leaving this organization.” Ratings were made on a 5-point response scale ranging from 1 (*does not agree*) to 5 (*fully agree*). Omega (ω) reliability of the scale was 0.934, 95% bootstrap CI [0.923,0.944].

#### Burnout

Burnout was assessed with a 5-item scale (Pejtersen et al., 2010). An example item is “felt worn out,” and rated on a five-point response scale ranging from 1 (*not at all*) to 5 (*the whole time*). Omega (ω) reliability of the scale was 0.903, 95% bootstrap CI [0.893,0.912].

#### Work Role Performance

Work role performance was assessed with a 3-item individual task proficiency scale (Griffin et al., 2007). An example item is “Carried out the core parts of your job well.” Ratings were made on a 5-point response scale ranging from 1 (*to a very low extent*) to 5 (*a very large extent*). Omega (ω) reliability of the scale was 0.847, 95% bootstrap CI [0.818,0.870].

#### Role Clarity

Role clarity was assessed with a 3-item scale (Pejtersen et al., 2010). An example item is “Does your work have clear objectives?” Ratings were made on a 5-point scale ranging from 1 (*to a very low extent*) to 5 (*to a very large extent*). Omega (ω) reliability of the scale was 0.869, 95% bootstrap CI [0.850,0.885].

#### Job Satisfaction

Job satisfaction was assessed with a single item: “How pleased are you with your job as a whole, everything taken into consideration?” (Pejtersen et al., 2010). Ratings were made on a 5-point scale raining from 1 (*to a very low extent*) to 5 (to *a very high extent*).

### Analysis

#### Factor Structure of Destrudo-L

To address research question 1 and 2, we estimated ICM-CFA, ESEM, and bifactor models to identify the best fitting model. We used Mplus version 8.4 (Muthén and Muthén, 1998–2017) and the robust means- and variance-adjusted weighted least squares estimator (WLSMV; Finney and DiStefano, 2013) to estimate the ICM-CFA, ESEM, and bifactor models. We used target rotation (Browne, 2001; Asparouhov and Muthén, 2009) in the ESEM models, which allows for the specification of factor loadings on target and non-target latent factors in a confirmatory manner. All cross-loadings were specified as being close to zero but not exactly zero, whereas the main factor loadings were freely estimated (Morin et al., 2016). In the ICM-CFA, all cross-loadings were fixed to zero.

The bifactor models were specified with a global destructive leadership factor, alongside five specific factors representing the subdimensions of destructive leadership according to the bifactor ESEM framework recently proposed by Morin et al. (2016). The global factor explains variance shared across all items and the specific factors in bifactor models explains item variance unaccounted for by the global factor. The specific and global factors were specified as orthogonal to ensure interpretability and adherence to bifactor assumptions (Chen et al., 2006; Reise, 2012).

Model fit was evaluated with conventional fit indices, such as the comparative fit index (CFI), the Tucker-Lewis index (TLI), the standardized root mean residual (SRMR), and the root mean square error of approximation (RMSEA). CFI and TLI values around 0.90, and SRMR and RMSEA values around 0.08, respectively indicated acceptable model fit (Marsh, 2007). It is important to remember that these are all rough guidelines, not “golden rules” (Marsh et al., 2004), and developed within a CFA framework. The extent to which they are relevant for ESEM applications is still unclear (Marsh et al., 2009b). Omega reliability coefficients (ω) and bootstrap confidence intervals were calculated based on the approximate and closed-form solution proposed by Hancock and An (2020).

To address research question 2, we examined latent variable correlations between the dimensions of Destrudo-L and leadership measures of abusive supervision and transformational leadership, as well as the employe outcome measures of OCB, turnover intention, burnout, work role performance, role clarity, and job satisfaction.

#### Destructive Leadership Profiles

To address research question 3 and 4, we performed latent profile analysis (LPA). Latent factor scores were saved and used as the input variables in the LPA to make sure the input variables represented true combinations of destructive leadership, and not shared error variance. Nested model comparisons where conducted, where more parsimonious models with fewer profiles were compared to more complex models with more profiles. Models with one to eight profiles were tested in the present study to identify the optimal number of profiles. We used several criteria to determine the optimal number of profiles (e.g., Nylund et al., 2007; Marsh et al., 2009a; Morin and Marsh, 2015), and relied primarily on four tests and indices that simulation studies have found particularly effective for model selection in LPA (see Yang, 2006; Henson et al., 2007; Nylund et al., 2007; Peugh and Fan, 2013). These four are the consistent Akaike’s information criterion (CAIC), Bayesian information criterion (BIC), the sample-size adjusted BIC (SSA-BIC), and the bootstrapped likelihood ratio test (BLRT). A better model fit is indicated by lower CAIC, BIC, and SSA-BIC values, and a statistically significant BLRT test (*p* < 0.05) indicates that the target profile solution fits better than a solution with one fewer profile. We also examined the entropy criterion, which varies from 0 to 1 and indicates how accurately people are categorized into their respective profiles. Higher entropy values indicate a better fit for a given solution (Aldridge and Roesch, 2008). Although the entropy criterion is considered a useful tool to assess classification accuracy (Morin and Marsh, 2015), it should not be used to determine the optimal number of profiles (Lubke and Muthén, 2007; Tein et al., 2013). In addition to the fit criteria, interpretability, theoretical meaningfulness, and parsimony of the latent profiles are also important to assess when determining the optimal solution (Muthén, 2003; Marsh et al., 2009a).

To aid the interpretation of the results, *z*-scores with a mean of 0 and standard deviation of 1 were used. Wald tests (the BCH method; Asparouhov and Muthén, 2020) were used for overall and pairwise comparisons to examine differences between the latent profiles on the outcome variables (i.e., turnover intentions, burnout, organizational citizenship behaviors, work-role performance, role clarity, and job satisfaction). We used Mplus version 8.4 (Muthén and Muthén, 1998–2017) and the robust full information maximum likelihood estimator (MLR) to estimate the LPA.

#### Prevalence

In addition to reporting on prevalence of any kind of destructive leadership behaviors (comparable to Schat et al., 2006), we also used two other commonly employed methods to estimate prevalence rates to address research question 5 and 6. First, we used the operational classification method (OCM), which defines a specific cut-off criterion to classify respondents as exposed or not exposed to destructive leadership (Aasland et al., 2010). As destructive leadership, by definition, is present only when repeatedly demonstrated (Einarsen et al., 2007; Schyns and Schilling, 2013), Aasland et al. (2010) applied a cut-off criterion of exposure to one or more destructive leadership behaviors “quite often” or more frequently. Hubert and van Veldhoven (2001), on the other hand, suggested a milder cut-off criterion of “sometimes” or more frequently. To enable comparisons with both of these studies, we used cut-off criteria at all levels, for overall destructive leadership (i.e., including all 20 items of Destrudo-L), as well as for all of the separate subscales. Additionally, we compared prevalence in the private and the public sectors using the stricter cut-off criterion “often.”

Second, although OCM is a common method of reporting prevalence rates, it has been criticized for reducing prevalence of exposure to a simple yes-or-no question. Also, it neither takes into account the number of items used, nor considers being exposed to only one form of behavior at a certain level to be sufficient (Aasland et al., 2010). Aasland et al. (2010) therefore suggested complementary use of latent class/profile analysis to determine prevalence. We instead use the results of the above presented LPA as an alternative way to determine prevalence. Going beyond Aasland et al. (2010) way of determining prevalence based on profiles and based on Shaw et al. (2011) reasoning, we included the global destructive leadership factor as an indicator of the leader being perceived as a destructive leader. We used *z*-score results below 0.5 as the cut-off for infrequently destructive leader, above 0.5 for moderately frequent destructive leader, and results above 1.0 for highly frequent destructive. To determine the validity of these cut-offs, we used analysis of the latent profiles association with outcomes (presented above).

## Results

### Descriptive Statistics and Preliminary Analysis

Descriptive statistics of the items and omega reliability coefficients are presented in Table 1. All items were positively skewed and had a non-normal response pattern, which shows the importance of using a robust estimator in subsequent analyses. Reliability coefficients ranged from 0.88 to 0.91 and the bootstrap confidence intervals were all narrow, indicating a relatively high precision of the point estimate. Following recommendations in the literature (Marsh et al., 2009b), we first compared the model fit of an ICM-CFA model with that of the ESEM. As seen in Table 2, the ESEM showed a better fit to the data, as indicated by a lower chi-square, higher CFI and TLI, and lower RMSEA and SRMR. The latent factor correlations (see Supplementary Table 1) were lower in the ESEM, ranging from 0.41 to 0.80 (*M* = 0.62), compared to the ICM-CFA, in which they ranged from 0.66 to 0.90 (*M* = 0.81). A comparison of the bifactor ICM-CFA and the bifactor ESEM also showed a superior model fit of the bifactor ESEM (Table 2).

**TABLE 1:** Item descriptives and reliability coefficients (ω) with 95% bootstrap confidence intervals.

	***N***	**Min**	**Max**	***M***	***SD***	**Skew.**	**Kurt.**	**ω**	**95% CI**
**Arrogant/Unfair**								0.913	[0.902,0.922]
Makes subordinates stupid	1113	1	6	1.87	1.30	1.33	0.57		
Behaves arrogant	1115	1	6	2.01	1.41	1.18	0.12		
Treats people differently	1109	1	6	2.86	1.62	0.36	–1.14		
Is unpleasant	1117	1	6	1.76	1.14	1.48	1.43		
**Threats/Punishments/Overdemands**								0.875	[0.855,0.893]
Shows violent tendencies	1118	1	6	1.61	1.10	1.89	2.87		
Punishes subordinates who makes mistakes or do not reach set goals	1113	1	6	1.58	1.08	2.07	3.81		
Uses threats to get his/her way	1114	1	6	1.44	0.99	2.48	5.80		
Puts unreasonable demands	1117	1	6	1.93	1.26	1.24	0.55		
**Ego-oriented/False**								0.889	[0.873,0.902]
Takes the honor of subordinates’ work	1111	1	6	1.83	1.28	1.48	1.17		
Puts own needs ahead of the group’s	1112	1	6	2.03	1.41	1.18	0.22		
Does not trust his/her subordinates	1117	1	6	1.99	1.33	1.24	0.53		
Does not keep promises	1115	1	6	2.11	1.32	0.99	–0.03		
**Passive/Cowardly**								0.885	[0.870,0.896]
Does not dare to confront others	1116	1	6	2.44	1.54	0.73	–0.71		
Does not show up among subordinates	1118	1	6	2.14	1.45	1.02	–0.25		
Does not show an active interest	1119	1	6	2.10	1.35	1.04	–0.01		
Does not take a grip on things	1119	1	6	2.40	1.50	0.71	–0.78		
**Uncertain/Unclear/Messy**								0.906	[0.896,0.916]
Shows insecurity in his/her role	1116	1	6	2.38	1.52	0.72	–0.78		
Is bad at structuring and planning	1116	1	6	2.55	1.52	0.61	–0.83		
Gives unclear instructions	1117	1	6	2.68	1.52	0.43	–1.02		
Behaves confused	1117	1	6	2.09	1.36	1.11	0.22		

**TABLE 2:** Model Fit Indices of the CFA, Bifactor CFA, ESEM, and Bifactor ESEM models.

	**χ ^2^**	**df**	***p***	**CFI**	**TLI**	**RMSEA**	**90% CI**	**SRMR**
CFA	1401.097	160	0.000	0.977	0.973	0.083	[0.079,0.087]	0.031
Bifactor CFA	2652.719	150	0.000	0.954	0.941	0.122	[0.118,0.126]	0.059
ESEM	484.278	100	0.000	0.993	0.986	0.059	[0.053,0.064]	0.011
Bifactor ESEM	315.663	85	0.000	0.996	0.990	0.049	[0.043,0.055]	0.009

*N = 1121.*

### Bifactor Structure of Destrudo-L

To address research question 1, we examined the factor loadings of the bifactor ESEM model (Table 3). The results indicated a well-defined global destructive leadership factor with standardized factor loadings (*λ*) ranging from 0.69 to 0.88 (*M* = 0.80). The standardized target factor loadings (*λ*) for the specific factors were weaker than those of the global factor, but they– still indicated relatively well-defined factors (arrogant/unfair, *λ* range 0.29–0.45, *M* = 0.37; threats/punishments/overdemands, *λ* range 0.25–0.42, *M* = 0.37; Ego/False, *λ* range −0.11 to 0.45, *M* = 0.16; passive/cowardly, *λ* range 0.23–0.58, *M* = 0.40; and uncertain/unclear/messy, *λ* range 0.31–0.52, *M* = 0.44), with few cross-loadings larger than 0.30. Overlap was most evident between the arrogant/unfair factor and threats/punishments/overdemands factor, where one item per subdimension (i.e., “Is unpleasant”; “Shows violent tendencies”) showed cross-loadings around 0.30. The ego-oriented/false subdimension was the least well-defined of the five subdimensions, with two items (i.e., “Does not trust his/her subordinates”; “Does not keep promises”) showing low and not statistically significant factor loadings on the target factor. To summarize, we were able to replicate the original bifactor structure of Destrudo-L using an ESEM approach, which provided a better fit to the data than the traditional ICM-CFA.

**TABLE 3:** Factor loading pattern of the bifactor ESEM.

	**AU**	**TPO**	**EF**	**PC**	**UUM**	**Global DL**	**Residual variance (1-*R*^2^)**
Makes subordinates stupid	0.363*	0.035	0.055*	−0.065*	−0.041*	0.823*	0.181
Behaves arrogant	0.446*	0.061*	0.040*	−0.033*	−0.067*	0.852*	0.065
Treats people differently	0.287*	0.066*	–0.031	–0.027	0.007	0.812*	0.252
Is unpleasant	0.390*	0.307*	−0.040*	–0.035	–0.004	0.802*	0.108
Shows violent tendencies	0.292*	0.432*	−0.050*	−0.049*	0.015	0.765*	0.138
Punishes subordinates who make mistakes or do not reach set goals	0.126*	0.363*	0.035	−0.076*	−0.125*	0.804*	0.183
Uses threats to get his/her way	0.024	0.416*	0.051*	−0.137*	−0.083*	0.828*	0.113
Puts unreasonable demands	–0.010	0.252*	0.113*	−0.107*	–0.036	0.769*	0.319
Takes the honor of subordinates’ work	–0.013	0.078*	0.447*	−0.065*	–0.033	0.826*	0.108
Puts own needs ahead of the group’s	0.025	0.026	0.267*	0.084*	–0.033	0.870*	0.162
Does not trust his/her subordinates	–0.008	0.064*	**0.024**	–0.031	−0.118*	0.880*	0.206
Does not keep promises	−0.113*	0.023	−0.108*	0.094*	0.043*	0.870*	0.207
Does not dare to confront others	−0.113*	−0.325*	−0.087*	0.227*	0.143*	0.737*	0.258
Does not show up among subordinates	–0.014	–0.037	0.024	0.395*	0.061*	0.690*	0.362
Does not show an active interest	0.014	–0.002	0.051*	0.581*	0.084*	0.779*	0.046
Does not take a grip on things	−0.095*	−0.112*	−0.096*	0.383*	0.230*	0.789*	0.148
Shows insecurity in his/her role	−0.069*	−0.230*	–0.032	0.202*	0.308*	0.740*	0.258
Is bad at structuring an planning	−0.034*	0.007	–0.023	0.135*	0.518*	0.768*	0.121
Gives unclear instructions	–0.024	–0.024	–0.008	0.118*	0.493*	0.781*	0.131
Behaves confused	0.016	–0.008	–0.012	0.008	0.430*	0.725*	0.289

AU, arrogant/unfair; TPO, threats/punishments/overdemands; EF, ego-oriented/false; PC, passive/cowardly; UUM, uncertain/unclear/messy; Global DL, global destructive leadership. *p < 0.05. Bold indicates the items included in scale.

To address research question 2, we estimated latent factor correlations between the destructive leadership factors and the outcome variables (Table 4). We found a clear pattern of associations between the global destructive leadership factor and the outcome variables. We observed positive correlations between global destructive leadership and the negative outcomes (i.e., turnover intentions and burnout), whereas the correlations between global destructive leadership and the positive outcomes (i.e., organizational citizenship behaviors, work-role performance, role clarity, job satisfaction) were negative. The global destructive leadership factor also had a negative correlation with transformational leadership and a positive correlation with abusive supervision. Most of the correlations between the specific destructive leadership factors and the outcome variables were small (*r*s < 0.30). The latent factor correlations, using a variable-centered approach, supports the convergent validity of the global destructive leadership factor, whereas the correlations with specific factors provide weak support for their convergent validity.

**TABLE 4:** hypLatent factor correlations between the specific and global destructive leadership factors and outcomes.

	**TOI**	**BO**	**OCB**	**WRP**	**RC**	**JS**	**TL**	**AS**
AU	0.104*	0.043	0.149*	–0.043	0.042	−0.098*	−0.210*	0.151*
TPO	–0.044	−0.154*	0.006	0.013	0.095*	0.038	0.067*	0.181*
EF	−0.130*	−0.316*	0.128*	0.141*	0.159*	0.085*	0.039	–0.052
PC	0.017	−0.125*	–0.073	0.058	–0.030	–0.017	−0.230*	–0.026
UUM	–0.035	−0.100*	–0.012	–0.022	−0.142*	–0.023	−0.128*	–0.034
Global DL	0.473*	0.430*	−0.095*	−0.111*	−0.408*	−0.347*	−0.804*	0.881*

AU, arrogant/unfair; TPO, threats/punishments/overdemands; EF, ego-oriented/false; PC, passive/cowardly; UUM, uncertain/unclear/messy; Global DL, global destructive leadership; OCB, organizational citizenship behaviors; TOI, turnover intentions; BO, burnout; WRP, work-role performance; RC, role clarity; JS, job satisfaction; TL, transformational leadership; AS, abusive supervision. *p < 0.05.

### Destructive Leadership Profiles

Based on the latent factor scores from the bifactor ESEM, we estimated a series of nested LPA models with increasing complexity (i.e., more profiles) and compared the model fit between these nested models to answer research question 3. Thus, the LPA input variables included the specific subdimensions and the global destructive leadership factor (cf. Morin et al., 2017). As seen in Table 5, the model fit improved for each added class. However, the 7-profile solution was chosen as the final model, because adding a seventh profile provided a theoretically intelligible and meaningful additional class, compared to a model with six profiles. Adding an eighth profile, however, resulted in an arbitrary division of an existing profile into smaller profiles that differed mostly in a quantitative way from each other. Further, the eighth class was small (∼ 4% of the sample) and did not add anything theoretically meaningful.

**TABLE 5:** Fit indices of the latent profile analysis based on the bifactor ESEM with global and specific factors.

**Model**	**LL**	**#fp**	**AIC**	**CAIC**	**BIC**	**ABIC**	**Entropy**	**BLRT**
1 Profile	−9543.781	12	19111.561	19183.825	19171.825	19133.710	na	
2 Profiles	−9114.505	19	18267.009	18381.427	18362.427	18302.078	0.776	0.000
3 Profiles	−8833.433	26	17718.866	17875.437	17849.437	17766.854	0.823	0.000
4 Profiles	−8718.258	33	17502.515	17701.241	17668.241	17563.424	0.827	0.000
5 Profiles	−8648.456	40	17376.911	17617.790	17577.790	17450.739	0.824	0.000
6 Profiles	−8592.803	47	17279.605	17562.638	17515.638	17366.353	0.823	0.000
**7 Profiles**	−**8254.671**	**54**	**17157.342**	**17482.529**	**17428.529**	**17257.010**	**0.819**	**0.000**
8 Profiles	−8452.440	61	17026.880	17394.221	17333.221	17139.468	0.843	0.000

*LL, Model loglikelihood; #fp, number of free parameters; SF, scaling factor of the robust Maximum Likelihood estimator; AIC, Akaike Information Criterion; CAIC, Consistent AIC; BIC, Bayesian Information Criterion; ABIC, sample-size Adjusted BIC; BLRT, Bootstrap Likelihood Ratio Test. Bold indicates the 7-profile solution, which is the final model.*

**FIGURE 1:**
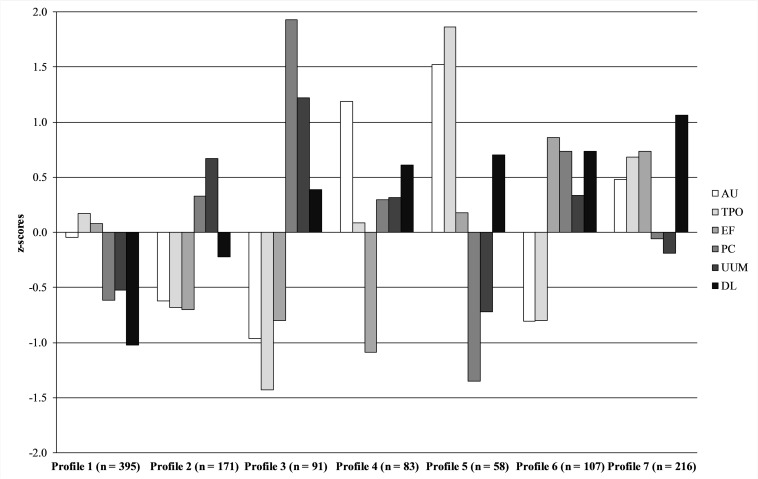
Destructive leadership profiles. The figure shows *z*-scores (*M* = 0, *SD* = 1). AU, arrogant/unfair; TPO, threats/punishments/overdemands; EF, ego-oriented/false; PC, passive/cowardly; UUM, uncertain/unclear/messy; DL, global destructive leadership factor.

The following is a description of the seven latent profiles (see Figure 1). Following previous studies (e.g., Gustafsson et al., 2018), the descriptions are based on standardized *z*-scores and represent standard deviation (*SD*) units above (i.e., positive values) or below (i.e., negative values) the sample mean (which is 0). We define larger than ± 1 *SD* as very low/high, ±0.5 to 1.0 *SD* as low/high, and values from -0.5 to 0.5 *SD* as slightly below/above average.

Profile 1 was labeled *consistently non-destructive* and characterized by average to very low scores on all destructive leadership behaviors. Profile 2 was labeled *somewhat messy* and characterized by low scores on the active behaviors (i.e., arrogant/unfair, threats/punishments/overdemands and ego-oriented/false) and high scores on the specific subdimension uncertain/unclear/messy. Profile 3 was labeled *passive* and characterized by low scores on the active behaviors and very high scores on the passive behaviors (i.e., passive/cowardly and uncertain/unclear/messy). Profile 4 was labeled *unfair-destructive* and characterized by high scores on the specific subdimension arrogant/unfair, low scores on the ego-oriented/false factor, as well as a *z*-score above 0.5 on the global destructive factor. Profile 5 was labeled *active destructive* and was characterized by very high scores on the active subdimensions arrogant/unfair and threats/punishments/overdemands, and low to very low on the passive forms, with a *z*-score above 0.5 on the global destructive factor. Profile 6 was labeled *ego-oriented passive destructive* and was characterized by high scores on the Ego-oriented/False factor and on passive destructive behaviors, as well as low scores on the active subdimensions arrogant/unfair and threats/punishments/overdemands. *Z*-score for the global destructive factor was again above 0.5. Profile 7 was labeled *ego-oriented active destructive* and characterized by very high scores (i.e., *z-*score ≥1.0) on the global destructive factor and high scores on the active behaviors.

**FIGURE 2:**
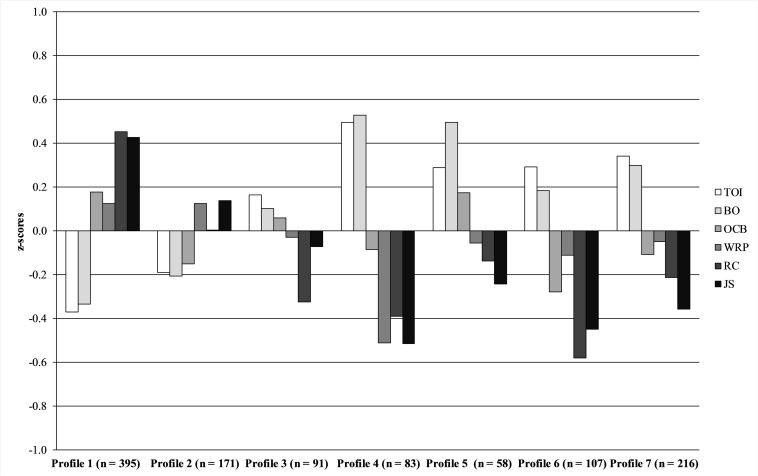
Differences between the destructive leadership profiles on the outcome variables. The figure shows *z*-scores (*M* = 0, *SD* = 1). TOI, turnover intentions; BO, burnout; OCB, organizational citizenship behaviors; WRP, work-role performance; RC, role clarity; JS, job satisfaction.

Comparisons between the latent profiles on the outcome variables (i.e., turnover intentions, burnout, organizational citizenship behaviors, work-role performance, role clarity, and job satisfaction), to answer research question 4, are presented in Supplementary Table 2 in the Supplementary Material and in Figure 2. Due to the large number of comparisons, the significance level was set to 1%, hence, a *p*-value lower than 0.01 was considered statistically significant. The omnibus Wald test indicated statistically significant differences between the profiles on all outcome variables (*p* < 0.005).

Pairwise comparisons showed that the consistently non-destructive profile (Profile 1) reported lower turnover intentions and burnout scores compared to all other profiles except the somewhat messy profile (Profile 2). Profile 1 also reported higher levels of organizational citizenship behaviors compared to the messy (Profile 2), ego-oriented passive destructive (Profile 6), and ego-oriented active destructive (Profile 7) profiles, higher work-role performance than the unfair destructive profile (Profile 4), and higher role clarity and job satisfaction compared to all other profiles.

The somewhat messy profile (Profile 2) reported lower turnover intentions and burnout scores than the passive (Profile 3), unfair destructive (Profile 4), active destructive (Profile 5), ego-oriented passive destructive (profile 6), and the ego-oriented active destructive (Profile 7) profiles. The somewhat messy profile (Profile 2) also reported higher work-role performance than the unfair destructive profile (Profile 4), higher role clarity than the ego-oriented passive destructive (Profile 6), and higher job satisfaction than the active destructive (Profile 4), ego-oriented passive destructive (Profile 6), and the ego-oriented active destructive (Profile 7) profiles. No other statistically significant differences were observed between the profiles.

The consistently non-destructive profile (Profile 1) showed the most favorable profile in relation to the outcomes variables, closely followed by the somewhat messy profile (Profile 2). Both of these profiles were characterized by relatively low levels of destructive leadership. The remaining profiles, particularly Profiles 4 to 7, reported high levels of destructive leadership, and these profiles also showed a more negative pattern on the outcome variables.

### Prevalence

To address research question 5, we estimated the prevalence of destructive leadership behaviors reported by the weighted sample, both in numbers and percent (Table 6). For item-level prevalence and prevalence of behaviors exposed to for each subdimension using the OCM criterion “often,” see Supplementary Tables 3, 4 in the Supplementary Material. In total, 90.2% of the weighted Swedish workforce sample reported exposure to destructive leadership behaviors of any kind. Using the OCM criterion of “often” or more frequently, we found that 36.4% of our sample was exposed to at least one destructive leadership behavior. The prevalence of the different types of destructive leadership behaviors in our sample varied widely: 8.7% for threats/punishments/overdemands, 16.9% for ego-oriented/false, 21.0% for arrogant/unfair, 23.5% for passive/cowardly, and 23.9% for uncertain/unclear/messy.

**TABLE 6:** Prevalence and frequency of destructive leadership behaviors at work in Sweden.

	**Prevalence%/Estimated Population Total**					
	**Never**	**Very seldom**	**Seldom**	**Sometimes**	**Often**	**Always**
**All Destructive Leadership Behaviors^*a*^**	9,8	17,0	12,9	23,9	21,3	15,1
	303 254	528 451	398 491	741 490	661 558	467 038
*OCM-criteria’s*	−	90,2	73,2	60,0	36,4	15,1
	−	2 797 028	2 268 577	1 870 086	1 128 596	467 038
**Arrogant, Unfair**	26,0	21,8	13,2	18,0	13,1	7,9
	805 070	677 322	410 132	558 163	406 138	243 457
*OCM-criteria’s*	−	74,0	52,2	39,0	21,0	7,9
	−	2 295 212	1 617 890	1 207 758	649 595	243 457
**Threats, Punishments, Overdemands**	44,0	22,0	11,5	13,8	6,4	2,3
	1 362 868	682 665	355 124	428 549	198 603	72 473
*OCM-criteria’s*	−	56,0	34,0	22,6	8,7	2,3
	-	1 737 414	1 054 749	699 625	271 076	72 473
**Ego-oriented, False**	33,6	23,2	14,6	11,7	12,2	4,8
	1 041 998	719 786	452 382	361 360	377 248	147 508
*OCM-criteria’s*	−	66,4	43,2	28,6	16,9	4,8
	−	2 058 284	1 338 498	886 116	524 756	147 508
**Passive, Cowardly**	26,8	18,9	10,9	19,9	16,1	7,5
	831 324	584 835	337 828	616 285	498 199	231 811
*OCM-criteria’s*	−	73,2	53,3	43,4	23,5	7,5
	−	2 268 958	1 684 123	1 346 295	730 010	231 811
**Uncertain, Unclear, Messy**	22,0	21,4	11,5	21,2	17,2	6,6
	681 868	662 834	355 914	657 561	532 513	209 592
*OCM-criteria’s*	−	78,0	56,6	43,2	23,9	6,8
	−	2 418 414	1 755 580	1 399 666	742 105	209 592

*Estimated Population Total *N* = 3 100 282, Sample *N* = 1.132. ^*a*^All Destructive Leadership Behaviors includes all 20 items in the Destrudo-L questionnaire.*

Prevalence based on the results of the above presented LPA (Figure 1), with the global destructive leadership factor as an indicator of the leader being perceived as a destructive leader, yields somewhat different results. Using population weights: Profile 1 represented 34.9% (*N* = 1082047); Profile 2 represented 13.7% (*N* = 424952); Profile 3 represented 7.9% (*N* = 245521); Profile 4 represented 8.2% (*N* = 252537); Profile 5 represented 5.5% (*N* = 171685); Profile 6 represented 10.1% (*N* = 312314); and Profile 7 represented 19.7% (*N* = 611226). Thus, 23.8%, representing Profiles 4, 5, and 6, were considered as exposed to a moderately frequent (*z*-value above 0.5) destructive leader, and 19.7%, representing Profile 7, were considered as exposed to a highly frequent (*z*-value above 1.0) destructive leader. In total, 43.5% of the weighted sample was exposed to a moderate to highly frequent destructive leader. The association between the profiles and outcomes also show that these four profiles are most clearly associated with adverse outcomes (Figure 1).

Finally, to answer research question 6, we compared the private and public sectors (see Table 7), using the OCM criterion of “often” or more frequently, and found that exposure to destructive leadership behaviors of any kind was more common in the public sector (38.2%, compared to 35.1%, χ^2^ = 4727,95, *df* = 1, *p* < 0.001). Looking at the different types of destructive leadership behaviors, more active/direct forms were somewhat more frequent in the public sector, whereas passive/indirect destructive leadership was more frequent in the private sector.

**TABLE 7:** Prevalence and frequency of destructive leadership behaviors ‘often’ or ‘always’ (OCM criteria) in different sectors at work in Sweden.

		Prevalence%/Estimated Population Total			
	All Private	All Public	*Public- Municipality*	*Public- County*	*Public-State*
All Destructive Leadership Behaviors^a^	35,1	38,2	37,7	39,2	38,0
	629 867	498 729	104 022	113 330	281 377
Arrogant, Unfair	20,8	21,2	19,1	22,8	21,3
	373 649	275 901	52 592	65 927	157 382
Threats, Punishments, Overdemands	7,9	9,9	8,9	9,1	10,6
	142 191	128 885	24 475	26 371	78 039
Ego-oriented, False	19,0	14,0	14,1	15,3	13,5
	341 678	183 079	38 996	44 328	99 755
Passive, Cowardly	25,0	21,6	20,0	22,1	21,9
	448 666	281 343	55 196	63 804	162 343
Uncertain, Unclear, Messy	25,5	21,8	20,4	22,3	22,1
	458 000	284 105	56 298	64 490	163 317

*Estimated Population Total *N* = 3 100 282 (Total Sample *N* = 1.132), All Private *N* = 1 795 839 (218), All Public *N* = 1 304 443 (914), Public- Municipality *N* = 275 896 (329), Public- County *N* = 288 841 (312), Public-State *N* = 739 706 (273).*

*^*a*^All Destructive Leadership Behaviors includes all 20 items in the Destrudo-L questionnaire.*

*All private vs. All public on all DL – (χ^2^ = 4727,95, *df* = 1, *p* < 0.001).*

## Discussion

The present study set out to examine destructive leadership as a multidimensional construct using both variable- and person-centered approaches. The variable-centered analyses supported the structural validity of Destrudo-L, a multidimensional measure of destructive leadership, reflecting both a global factor and specific subdimensions. Convergent validity, in terms of associations with employe outcomes, however, were stronger for the global factor than the subdimensions. The person-centered analyses suggested seven distinct destructive leadership profiles. In addition to the global destructive leadership factor, they differed in their configurations of the subdimensions, with the consistently non-destructive and somewhat messy profiles showing a more favorable relation to employe outcomes such as well-being and work performance. Finally, we found that a substantial proportion of the Swedish workforce report being exposed to destructive leadership (36.4–43.5%, depending on type of analysis). Extending previous findings, our analyses suggest a somewhat higher prevalence in the public sector, compared to the private sector.

The examination of the factor structure of Destrudo-L (RQ1) showed that the best fit to the data was achieved with the bifactor ESEM model, which suggests that the factor structure of the Destrudo-L is more complex than reported in previous studies using ICM-CFA and bifactor ICM-CFA models (cf. Larsson et al., 2012). However, a commonality between our findings and previous findings is the determination that the Destrudo-L is best represented by a bifactor structure, simultaneously modeling a global destructive leadership factor and specific subdimensions. Although our results indicated a well-defined global destructive leadership factor, as demonstrated by the strong factor loadings on the global factor, several of the subdimensions were also relatively well-defined. These findings suggest that destructive leadership is a multidimensional construct, implying that previous unidimensional measures of destructive leadership or abusive supervision (e.g., Tepper, 2000) do not capture the construct in its full complexity. Hence, Destrudo-L overcomes some of the limitations of previous measures. However, one subdimension, ego-oriented/false, had items with weak and non-significant factor loadings on the specific factor. This was also the case in the original validation of Destrudo-L (cf. Larsson et al., 2012). One plausible explanation may be that two of the items are positively worded, whereas the other two are negatively worded, which likely introduced a methodological artifact, due to respondent carelessness (Roszkowski and Soven, 2010). Future studies should reformulate the items using either positively or negatively worded items to determine whether this issue can be resolved. Another possible explanation may be that ego-oriented and false are two distinct constructs and it is possible to question whether this is actually an active dimension of destructive leadership behaviors, as suggested by Larsson et al. (2012). This implies a need for further conceptual development of this specific subfactor, in addition to the reformulation of items.

The convergent and discriminant validity of Destrudo-L (RQ2), in terms of associations with other measures of constructive and destructive leadership, as well as with employe outcomes, were strongest for the global destructive leadership factor. The subdimensions had low and mostly non-significant correlations with the other constructs. A weak relation between the passive destructive leadership dimensions of Destrudo-L and the more active destructive behaviors measured in Tepper’s abusive supervision scale can perhaps be expected and supports the discriminant validity of Destrudo-L. However, beyond that, the minimally significant and non-significant correlations with subfactors may also reflect the limitations of using a variable-centered approach, where the global destructive leadership factor and the subdimensions are studied in isolation. These results highlight the need to specify measurement models that take different sources of construct-relevant multidimensionality into account (Morin et al., 2016). Given that our analysis of the factor structure of Destrudo-L demonstrated that destructive leadership is best understood as consisting of a global factor and subdimensions, this also needs to be reflected in the subsequent person-centered analyses.

We found seven profiles that yielded a meaningful differentiation of destructive leadership behaviors (RQ3): one characterized by non-destructive leadership, two by passive destructive, and four by different flavors of active destructive leadership. A global destructive leadership factor was included in all profiles. This provides a more realistic representation of destructive leadership: As suggested by the factor structure, the subdimensions are nuances of destructive leadership rather than isolated phenomena.

From this, it follows that the degree of global destructive leadership is one aspect of the disparity across the profiles, and may explain some of the differences in employe outcomes between profiles (RQ4). The consistently non-destructive and somewhat messy profiles, with very low scores on the global destructive factor, were the two profiles that differed the most, compared to the other profiles, in relation to employe outcomes. Beyond the global aspect, the seven profiles also seem to differ along a passive–active dimension, with the passive destructive leadership profiles being less harmful in relation to employe outcomes than the active. This was particularly evident for outcomes such as turnover intentions, burnout, and job satisfaction. Noteworthy is that although the passive destructive profiles seem less harmful than active destructive ones, they are still not equivalent to non-destructive. Thus supporting the previous suggestion that passive destructive leadership should be included in the definition of destructive leadership (Skogstad et al., 2007, 2014).

The findings in the current study are in line with those of Skogstad et al. (2015), who found that passive destructive leadership was less harmful for employe job satisfaction, in the short run, than active destructive leadership. However, they also found a cumulative effect, with passive destructive leadership being more harmful over time, relative to active destructive leadership. In addition, passive destructive leadership is more prevalent in both our and Aasland et al. (2010) studies. Consequently, these less severe but more prevalent leadership behaviors may, due to their commonality, have an equally profound impact on employes. More research is needed to disentangle the short- and long-term effects of active and passive destructive leadership on employes, as well as their impact, given their frequency in appearance.

Our study is the first to relate multidimensional destructive leadership profiles to employe outcomes, thereby providing a more detailed picture of how destructive leadership behavior may influence employe well-being and performance. Our findings show that the unfair destructive leadership profile reported lower work-role performance, whereas the ego-oriented passive destructive profile reported lower OCB and role clarity. These findings may be interpreted as a reflection of, in the first case, a leader who does not fairly acknowledge employe contributions, which is likely associated with reduced work performance among employes. In the second case, a passive destructive leader acting in an ego-oriented manner, may be perceived as not modeling OCB themselves, which may subsequently lead employes to follow suit. Passive destructive leadership behaviors may also, to a larger extent (than active destructive leadership), create role confusion, and thereby indirectly relate to unwanted employe outcomes such as burnout; this is a relation suggested by findings in previous leadership research (e.g., Vullinghs et al., 2018).

Regarding the prevalence of destructive leadership behaviors, we found that a substantial proportion of the Swedish workforce report being exposed to destructive leadership (RQ5). The exact number is highly dependent on the cut-off criteria and use of variable- or person-oriented approach. Over 90% of the Swedish workforce reported being exposed to at least one destructive leadership behavior, and around a third (36.4%) reported this happening often or always. Both numbers are slightly higher than those reported in the Norwegian study (83.5% overall exposure and 33.5% often or always, respectively), which is the only prevalence study that also combined multiple aspects of destructive leadership behaviors (Aasland et al., 2010).

However, the sum of the prevalence for the latent profiles that was 1/2 *SD* above the mean in global destructive leadership (Profiles 4–7) was 43.5%, which is considerably lower than the 61% in the Norwegian sample. This may be because of the criteria of 1/2 *SD*. Instead, summing the prevalence of all non-destructive profiles (2–7), in a way similar to Aasland et al. (2010) procedure, would yield a prevalence (65.1%), closer to theirs –61%. However, the results of the profiles’ association with outcomes in our study, where the non-destructive profiles (1–3) show a more favorable pattern compared to the destructive profiles (4–7), reveals, we believe, a more accurate picture of prevalence. Thus, we suggest that future prevalence studies should include the global destructive leadership dimension in the profile analysis, or use other means to determine perceived exposure (e.g., as suggested by Shaw et al., 2011; or Nielsen et al., 2009).

The most prevalent subdimensions of destructive leadership behaviors in our sample (per the OCM criterion) were the two passive dimensions (23.5% for passive/cowardly, and 23.9% for uncertain/unclear/messy), followed by arrogant/unfair (21.0%). The prevalence of threats/punishments/overdemands, which is the subdimension most similar to what previous studies have labeled manager aggression (Hubert and van Veldhoven, 2001; Schat et al., 2006), is experienced often or always by 8.7% by the population. Hubert and van Veldhoven (2001) used “at least sometimes” as a cut-off point, and with that criterion, and applying that criterion to our sample, the 22.6% we found is substantially higher than the 9% reported by Hubert and van Veldhoven (2001) and the 13.5% reported in the weighted U.S. sample (Schat et al., 2006). This may be due to differences in the constructs assessed, as well as operationalizations – for example, Hubert and van Veldhoven (2001) used a single item, whereas our subdimension included four items.

This study is, to the best of our knowledge, the first to compare the prevalence between the public and the private sector (RQ6). Although these represent small differences, destructive leadership behaviors were overall more common in the public sector. Active/direct forms of destructive leadership were more frequent in the public sector, and passive/indirect forms of destructive leadership more frequent in the private sector. This is in line with previous studies showing that highly active constructive leadership behaviors (i.e., a transformational leadership) is somewhat more common in the public sector, compared to the private sector (Lowe et al., 1996). Destructive leadership thus follows a similar pattern to that of constructive leadership (i.e., more active forms being more prevalent in public compared to private sector). Public sector leaders often lack possibilities to lead by incentives (a possibility given to a larger extent in the private sector), and are therefore more dependent on their own active leadership behaviors (An et al., 2019).

### Implications for Practice

The findings are relevant to organizations in a number of ways. First, our results offer additional evidence in support of the reliability and validity of Destrudo-L, suggesting that it can be used to assess employes’ perceptions of destructive leadership behaviors in a general work context. Second, our study demonstrates that destructive leadership is prevalent in all sectors of working life, and is therefore a potential problem that all organizations must address. Identifying destructive leaders, however, is not enough and in line with a framework recently developed by Pradhan and Jena (2018), we suggest that organizations take a broad approach in their efforts to reduce the prevalence of destructive leadership behaviors in organizations. This approach includes primary interventions, such as tailoring the recruitment and selection process to facilitate identification of personality traits that are associated with destructive leadership are identified. In addition, HR policies that reflect zero tolerance for abuse, as well as orientation programs in which these policies are explained to new leaders may be implemented. Secondary interventions include ensuring that the climate in the organization does not tolerate mistreatment or avoidance, as well as strategies to identify destructive leadership behaviors in the organizations, such as the inclusion of Destrudo-L in employes’ surveys, or establishing a HR hotline or other such complaint mechanisms. Finally, tertiary interventions may consist of counseling, problem solving and stress management, leadership training, withdrawal of benefits, and if nothing else works, termination. Intervention may also target employes in different ways, mitigating the negative effects of destructive leaders.

### Limitations and Future Research

This study is not without limitations. First, the data is cross-sectional. Although this is less of a problem in the context of investigating the factor structure (RQ1), profiles (RQ3), and prevalence (RQ5 and RQ6), it may be an issue for the interpretation of relations between destructive leadership and outcomes (RQ2 and RQ4). Particularly, Skogstad et al. (2014) found that, although active destructive leadership was worse than passive destructive leadership, in the short term (6 months), passive destructive leadership may build up to cause more adverse outcome over a longer period of time. Thus, we interpret the relations to outcomes with caution, primarily using them to enhance our understanding of the construct of destructive leadership. We recommend that future studies use experimental and longitudinal designs to examine the relations between destructive leadership and employe outcomes.

The Destrudo-L uses a 6-point response scale ranging from 1 (*never/almost never*) to 6 (*always*). This is in line with the ambition to capture the respondents’ general perception of the frequency stimuli. However, it also means that there is room for multiple interpretation of what constitutes “always” or “sometimes,” and the scale does not provide information on severity. There is a need for future studies that investigate how people interpret different response categories. Testing and implementing response scales that captures frequency as well as perceived severity is an additional opportunity in future studies.

This study relies on self-ratings, with destructive leadership being rated by employes. Although we used employes’ experience of their leaders, not leaders’ own ratings of their leadership behaviors, there is a risk of common-source bias, primarily affecting RQ2 and RQ4, which investigate relations between destructive leadership and outcomes. Using others’ ratings of leadership, such as employes’ ratings of their leader, as has been done here, is usually considered an advantage over self-ratings, as it minimizes the risk of self-serving bias. However, although ratings of others reflect the behavior of the rated, they may also be colored by the rater’s expectations, as well as by how much information they have on which base their judgment (Lornudd, 2015). In line with this, different raters have different experiences of the person they rate, and thus, additional raters have been shown to add to the variance explained (Oh and Berry, 2009). Thus, future studies, using multiple sources (e.g., supervisor-, employe- and self-ratings), are warranted.

The seven profiles are data-driven and exploratory, and must be replicated in future studies. This includes future studies to illuminate whether the profiles are representative for other working populations, which profiles may be considered primary or peripheral, and whether the profiles are stable over time. The culture in North/Western Europe differs somewhat from South/Eastern Europe and societies in other parts of the world. The difference is mainly found in terms of a lower power distance (i.e., the extent to which power in a society is equally/unequally shared), which in turn is reflected in the disapproval or endorsement of an autocratic leadership style (Koopman et al., 1999). Thus, the destructiveness of certain leadership behaviors may be interpreted in different ways depending on the specific societal culture where it is performed. Future studies should therefore strive to examine perceptions of destructive leadership in country contexts beyond North/Western Europe to advance our understanding of cross-cultural aspects of destructive leadership. We also encourage future studies where different subpopulations (e.g., different branches of work, older and younger employes) are compared.

## Conclusion

From a conceptual and an empirical perspective, the present study shows that destructive leadership is best understood as a multidimensional phenomenon, and should therefore be evaluated using multidimensional measures, such as Destrudo-L. The use of profile analysis can serve as a complementary approach to identify realistic representations of destructive leadership. We identified seven latent profiles, which differed along a passive and active continuum of destructive leadership behaviors. These profiles show different relations to employe outcomes, in that the active destructive leadership profiles are more detrimental than the passive profiles. On the other hand, the passive profiles are more prevalent. Thus, destructive leadership comes in many shapes and forms, and leaders acting destructively cannot be viewed as representing a mere fraction. Rather, as the results of our prevalence analysis show, these behaviors are frequently present at most places of work. Given the severe effects, and the commonness of these behaviors, organizations should work actively with strategies to identify and intervene, to prevent and to handle the appearance of these harmful behaviors.

## Data Availability Statement

The raw data supporting the conclusions of this article will be made available by the authors, without undue reservation.

## Ethics Statement

The studies involving human participants were reviewed and approved by the Regional Board of Ethics, Umeå (grant 2018/454-31). The patients/participants provided their written informed consent to participate in this study.

## Author Contributions

All authors provided substantial contributions to the conception or design of the work. ST was responsible for coordinating the data collection. RL drafted the introduction and performed the prevalence analysis. AS performed factor analysis and latent profile analysis. AS and RL drafted the result section. ST and UT drafted the discussion. All authors contributed during the interpretation process of the results, revising it critically for important intellectual content, and approved the final manuscript.

## Conflict of Interest

The authors declare that the research was conducted in the absence of any commercial or financial relationships that could be construed as a potential conflict of interest.

## Publisher’s Note

All claims expressed in this article are solely those of the authors and do not necessarily represent those of their affiliated organizations, or those of the publisher, the editors and the reviewers. Any product that may be evaluated in this article, or claim that may be made by its manufacturer, is not guaranteed or endorsed by the publisher.

## Supplementary Material

Supplementary Table 1 Latent factor correlations.

Supplementary Table 2 Class comparisons.

Supplementary Table 3 OCM Criterion.

Supplementary Table 4 Item-level prevalence.
