# Supplementary material for: Appetite for Destruction: A Psychometric Examination and Prevalence Estimation of Destructive Leadership in Sweden
Source: Front Psychol. 2021 Aug 6;12:668838. doi: 10.3389/fpsyg.2021.668838 (PMC8377166; doi:10.3389/fpsyg.2021.668838)
Supplement: Supplementary Table 1 — Latent factor correlations. [file Table_1.DOCX]

Table S1. Latent Factor Correlations of the ICM-CFA (above the diagonal), ESEM (below the diagonal)

|  | AU | TPO | EF | PC | UUM |
| --- | --- | --- | --- | --- | --- |
| AU |  | .936 | .886 | .721 | .727 |
| TPO | .773 |  | .905 | .660 | .687 |
| EF | .666 | .556 |  | .861 | .820 |
| PC | .618 | .411 | .644 |  | .900 |
| UUM | .631 | .451 | .624 | .800 |  |

*Note*. AU = arrogant/unfair, TPO = threats/punishments/overdemands, EF = ego-oriented/false, PC = passive/cowardly, UUM = uncertain/unclear/messy
