# Supplementary material for: Appetite for Destruction: A Psychometric Examination and Prevalence Estimation of Destructive Leadership in Sweden
Source: Front Psychol. 2021 Aug 6;12:668838. doi: 10.3389/fpsyg.2021.668838 (PMC8377166; doi:10.3389/fpsyg.2021.668838)
Supplement: Supplementary Table 2 — Class comparisons. [file Table_2.DOCX]

Table S2. Comparisons Between the Latent Profiles on the Outcome Variables

|  | Turnover  intentions | | Burnout | | Organizational  citizenship  behaviors | | Work-role  performance | | Role  clarity | | Job  satisfaction | |
| --- | --- | --- | --- | --- | --- | --- | --- | --- | --- | --- | --- | --- |
|  | χ^2^ | *p* | χ^2^ | *p* | χ^2^ | *p* | χ^2^ | *p* | χ^2^ | *p* | χ^2^ | *p* |
| Overall test | 109.627 | 0.000 | 96.772 | 0.000 | 23.868 | 0.001 | 18.381 | 0.005 | 141.191 | 0.000 | 140.869 | 0.000 |
| 1 vs. 2 | 2.514 | 0.113 | 1.372 | 0.241 | 7.505 | 0.006 | 0.000 | 0.996 | 17.683 | 0.000 | 7.971 | 0.005 |
| 1 vs. 3 | 18.064 | 0.000 | 9.865 | 0.002 | 0.821 | 0.365 | 1.415 | 0.234 | 33.199 | 0.000 | 13.108 | 0.000 |
| 1 vs. 4 | 31.548 | 0.000 | 34.408 | 0.000 | 2.895 | 0.089 | 13.279 | 0.000 | 30.404 | 0.000 | 45.376 | 0.000 |
| 1 vs. 5 | 16.633 | 0.000 | 26.408 | 0.000 | 0.000 | 0.992 | 1.021 | 0.312 | 10.651 | 0.001 | 13.140 | 0.000 |
| 1 vs. 6 | 24.033 | 0.000 | 20.252 | 0.000 | 13.668 | 0.000 | 3.365 | 0.067 | 51.322 | 0.000 | 47.018 | 0.000 |
| 1 vs. 7 | 61.729 | 0.000 | 43.654 | 0.000 | 8.393 | 0.004 | 3.690 | 0.055 | 56.901 | 0.000 | 64.530 | 0.000 |
| 2 vs. 3 | 4.895 | 0.027 | 3.442 | 0.064 | 1.552 | 0.213 | 0.926 | 0.336 | 3.882 | 0.049 | 1.595 | 0.207 |
| 2 vs. 4 | 14.423 | 0.000 | 18.853 | 0.000 | 0.125 | 0.724 | 10.413 | 0.001 | 4.873 | 0.027 | 15.943 | 0.000 |
| 2 vs. 5 | 7.214 | 0.007 | 16.249 | 0.000 | 3.155 | 0.076 | 0.894 | 0.344 | 0.527 | 0.468 | 3.739 | 0.053 |
| 2 vs. 6 | 8.410 | 0.004 | 7.464 | 0.006 | 0.630 | 0.427 | 2.242 | 0.134 | 11.378 | 0.001 | 14.348 | 0.000 |
| 2 vs. 7 | 19.869 | 0.000 | 18.507 | 0.000 | 0.116 | 0.734 | 2.262 | 0.133 | 3.435 | 0.064 | 16.978 | 0.000 |
| 3 vs. 4 | 3.208 | 0.073 | 5.084 | 0.024 | 0.582 | 0.445 | 5.697 | 0.017 | 0.116 | 0.734 | 5.667 | 0.017 |
| 3 vs. 5 | 0.442 | 0.506 | 3.911 | 0.048 | 0.356 | 0.551 | 0.018 | 0.893 | 0.755 | 0.385 | 0.588 | 0.443 |
| 3 vs. 6 | 0.542 | 0.462 | 0.227 | 0.634 | 3.900 | 0.048 | 0.236 | 0.627 | 1.750 | 0.186 | 4.227 | 0.040 |
| 3 vs. 7 | 1.743 | 0.187 | 1.683 | 0.195 | 1.303 | 0.254 | 0.022 | 0.881 | 0.589 | 0.443 | 3.343 | 0.067 |
| 4 vs. 5 | 0.945 | 0.331 | 0.023 | 0.879 | 1.501 | 0.221 | 3.694 | 0.055 | 1.233 | 0.267 | 1.526 | 0.217 |
| 4 vs. 6 | 1.129 | 0.288 | 4.088 | 0.043 | 1.110 | 0.292 | 3.994 | 0.046 | 0.913 | 0.339 | 0.137 | 0.712 |
| 4 vs. 7 | 0.801 | 0.371 | 1.929 | 0.165 | 0.015 | 0.903 | 6.157 | 0.013 | 1.086 | 0.297 | 0.928 | 0.335 |
| 5 vs. 6 | 0.000 | 0.988 | 2.947 | 0.086 | 5.704 | 0.017 | 0.073 | 0.786 | 4.063 | 0.044 | 0.965 | 0.326 |
| 5 vs. 7 | 0.090 | 0.764 | 1.246 | 0.264 | 2.398 | 0.122 | 0.001 | 0.969 | 0.143 | 0.706 | 0.316 | 0.574 |
| 6 vs. 7 | 0.107 | 0.744 | 0.699 | 0.403 | 1.370 | 0.242 | 0.194 | 0.660 | 5.070 | 0.024 | 0.368 | 0.544 |
