# Supplementary material for: Appetite for Destruction: A Psychometric Examination and Prevalence Estimation of Destructive Leadership in Sweden
Source: Front Psychol. 2021 Aug 6;12:668838. doi: 10.3389/fpsyg.2021.668838 (PMC8377166; doi:10.3389/fpsyg.2021.668838)
Supplement: Supplementary Table 3 — OCM Criterion. [file Table_3.DOCX]

Table S3. Exposure to Destructive leadership behaviors ‘often’ or ‘always’ (OCM-criteria)

|  | Number of Behaviors exposed to | | | | | |
| --- | --- | --- | --- | --- | --- | --- |
|  | | 0 | 1 | 2 | 3 | 4 |
| Arrogant, Unfair | | 79,0 | 10,9 | 3,7 | 4,1 | 2,2 |
|  | | 2 450 687 | 338 155 | 113 346 | 128 448 | 69 646 |
| Threats, Punishments, Overdemands | | 91,3 | 5,8 | 1,3 | 0,6 | 1,0 |
|  | | 2 829 206 | 179 277 | 40 233 | 18 505 | 33 062 |
| Ego-oriented, False | | 83,1 | 8,7 | 4,4 | 2,4 | 1,4 |
|  | | 2 575 526 | 268 708 | 137 279 | 73 983 | 44 786 |
| Passive, Cowardly | | 76,5 | 9,5 | 6,5 | 5,4 | 2,2 |
|  | | 2 370 272 | 295 060 | 200 157 | 166 871 | 67 922 |
| Uncertain, Unclear, Messy | | 76,1 | 8,6 | 6,8 | 4,9 | 3,6 |
|  | | 2 358 177 | 266 581 | 212 016 | 150 940 | 112 568 |

*Note.* Frequency in percent and estimated population total for rows (Estimated population total *N* = 3 100 282)
