# Supplementary material for: Appetite for Destruction: A Psychometric Examination and Prevalence Estimation of Destructive Leadership in Sweden
Source: Front Psychol. 2021 Aug 6;12:668838. doi: 10.3389/fpsyg.2021.668838 (PMC8377166; doi:10.3389/fpsyg.2021.668838)
Supplement: Supplementary Table 4 — Item-level prevalence. [file Table_4.DOCX]

Table S4. Prevalence and Frequency of Destructive Leadership Behaviors at Work in Sweden

|  |  |  |  |  |  |  |
| --- | --- | --- | --- | --- | --- | --- |
|  | Prevalence % / Estimated Population Total | | | | | |
| Type of Destructive Leadership Behavior | Never | Very seldom | Seldom | Sometimes | Often | Always |
| 1. Makes subordinates stupid | 55,7 | 20,2 | 6,0 | 11,1 | 4,2 | 2,8 |
|  | 1 725 622 | 625 218 | 185 578 | 345 110 | 130 935 | 87 818 |
| 2. Behaves arrogant | 56,7 | 15,4 | 6,8 | 12,4 | 5,3 | 3,4 |
|  | 1 757 524 | 478 216 | 211 530 | 384 289 | 164 132 | 104 585 |
| 3. Treats people differently | 28,8 | 20,4 | 13,8 | 17,1 | 12,9 | 7,1 |
|  | 893 108 | 631 178 | 427 009 | 530 349 | 398 437 | 219 201 |
| 4. Is unpleasant | 60,2 | 18,9 | 8,1 | 8,9 | 1,9 | 2,1 |
|  | 1 865 055 | 585 816 | 249 942 | 275 802 | 57 984 | 65 683 |
| 5. Shows violent tendencies | 68,7 | 14,6 | 8,1 | 5,4 | 2,0 | 1,2 |
|  | 2 130 448 | 452 128 | 250 998 | 167 397 | 62 476 | 35 843 |
| 6. Punishes subordinates who makes mistakes or do not reach set goals | 69,9 | 15,6 | 6,2 | 5,4 | 1,3 | 1,5 |
|  | 2 167 376 | 482 993 | 192 537 | 168 723 | 41 586 | 47 067 |
| 7. Uses threats to get his/her way | 77,7 | 10,8 | 4,0 | 5,2 | 0,9 | 1,3 |
|  | 2 409 156 | 336 241 | 124 707 | 160 191 | 28 841 | 41 145 |
| 8. Puts unreasonable demands | 53,3 | 20,3 | 9,9 | 10,4 | 5,0 | 1,2 |
|  | 1 653 199 | 629 122 | 306 927 | 321 559 | 153 689 | 35 866 |
| 9. Takes the honor of subordinates’ work | 59,6 | 17,5 | 8,5 | 7,4 | 4,9 | 2,0 |
|  | 1 849 175 | 543 163 | 264 343 | 228 182 | 152 790 | 62 628 |
| 10. Puts own needs ahead of the group’s | 54,3 | 18,2 | 9,4 | 9,5 | 6,1 | 2,4 |
|  | 1 683 413 | 563 565 | 292 491 | 294 674 | 190 525 | 75 613 |
| 11. Does not trust his/her subordinates | 53,5 | 18,6 | 10,5 | 10,8 | 4,5 | 2,1 |
|  | 1 657 417 | 576 717 | 326 438 | 335 647 | 138 378 | 65 695 |
| 12. Does not keep promises | 47,2 | 23,8 | 11,6 | 9,1 | 6,5 | 1,8 |
|  | 1 462 912 | 737 528 | 358 125 | 282 986 | 201 857 | 56 875 |
| 13. Does not dare to confront others | 40,7 | 19,9 | 10,4 | 16,2 | 8,4 | 4,4 |
|  | 1 262 911 | 616 687 | 322 583 | 502 315 | 260 869 | 134 918 |
| 14. Does not show up among subordinates | 51,4 | 17,4 | 11,2 | 8,8 | 9,4 | 1,9 |
|  | 1 593 737 | 539 034 | 347 989 | 271 489 | 289 907 | 58 127 |
| 15. Does not show an active interest | 49,5 | 19,6 | 10,6 | 10,1 | 8,6 | 1,6 |
|  | 1 536 070 | 606 872 | 328 683 | 313 885 | 266 105 | 48 666 |
| 16. Does not take a grip on things | 39,5 | 20,6 | 9,0 | 17,7 | 9,5 | 3,7 |
|  | 1 225 026 | 639 735 | 279 058 | 547 382 | 295 194 | 113 887 |
| 17. Shows insecurity in his/her role | 44,6 | 19,3 | 9,4 | 15,2 | 8,3 | 3,3 |
|  | 1 382 913 | 597 539 | 291 098 | 470 034 | 256 684 | 102 014 |
| 18. Is bad at structuring and planning | 32,5 | 22,6 | 13,5 | 14,4 | 12,8 | 4,2 |
|  | 1 007 983 | 702 035 | 419 194 | 445 816 | 395 295 | 129 961 |
| 19. Gives unclear instructions | 30,3 | 21,9 | 15,1 | 18,0 | 11,4 | 3,3 |
|  | 938 247 | 678 262 | 469 646 | 557 407 | 354 873 | 101 849 |
| 20. Behaves confused | 47,9 | 22,0 | 11,5 | 10,4 | 4,9 | 3,3 |
|  | 1 484 587 | 681 816 | 357 135 | 323 714 | 151 035 | 101 994 |

*Note*. Estimated population *N* = 3 100 282
